# Geographic Distribution and Risk Factors of the Initial Adult Hospitalized Cases of 2009 Pandemic Influenza A (H1N1) Virus Infection in Mainland China

**DOI:** 10.1371/journal.pone.0025934

**Published:** 2011-10-12

**Authors:** Yunning Liu, Wei Wang, Xia Li, Hong Wang, Yanxia Luo, Lijuan Wu, Xiuhua Guo

**Affiliations:** 1 Department of Epidemiology and Health Statistics, School of Public Health and Family Medicine, Capital Medical University, Beijing, China; 2 Beijing Municipal Key Laboratory of Clinical Epidemiology, Beijing, China; University of Nebraska – Lincoln, United States of America

## Abstract

**Background:**

As of 31^st^ March 2010, more than 127,000 confirmed cases of 2009 pandemic influenza A (H1N1), including 800 deaths, were reported in mainland China. The distribution and characteristics of the confirmed cases in the initial phase of this pandemic in this country are largely unknown. The present study aimed to characterize the geographic distribution and patient characteristics of H1N1 infection in the 2009 pandemic as well as to identify potential risk factors associated with adverse patient outcome in China, through retrospective analyses of 885 hospitalized cases with confirmed H1N1 infection.

**Methodology/Principal Findings:**

The proportional hazards model was employed to detect risk factors for adverse outcome; the geo-statistical maps were used to characterize the distribution of all 2668 confirmed H1N1 patients throughout mainland China. The number of new cases increased slowly in May, 2009, but rapidly between June and August of the year. Confirmed cases were reported in 26 provinces; Beijing, Guangdong, Shanghai, Zhejiang and Fujian were the top five regions of the incidence of the virus infection. After being adjusted for gender, age, chronic pulmonary disease and other general symptoms, delay for more than two days before hospital admission (HR: 0.6; 95%CI: 0.5–0.7) and delayed onset of the H1N1-specific respiratory symptoms (HR: 0.3; 95%CI: 0.2–0.4) were associated with adverse patient outcome.

**Conclusions/Significance:**

The 2009 pandemic influenza A affected east and southeast coastal provinces and most populous cities more severely than other regions in mainland China due to higher risk of high level traffic-, high population density-, and high population mobility-associated H1N1 transmission.The clinical symptoms were mild in the initial phase of infection. Delayed hospital admission and delayed appearance of respiratory symptoms were among the major risk factors for poor patient outcome. These findings may have significant implications in the future pandemic preparedness and response.

## Introduction

The 2009 pandemic influenza A (H1N1) was first reported in Mexico and then rapidly spread around the world, unfortunately due to the ‘convenience’ of airplane-based modern transportation system. On 11^th^ June 2009, the World Health Organization (WHO) raised the warning level to the sixth phase, indicating that the episode of influenza had entered a pandemic stage [1]. As of 11^th^ April 2010, more than 214 countries and territories or communities had reported laboratory-confirmed cases of 2009 H1N1, including over 17798 deaths [2]. The first case in mainland china was reported on May 11^th^, 2009. The Ministry of Health of the People's Republic of China released Guidelines for H1N1 Influenza Diagnosis and Treatment in the middle of July, 2009 [3]. By March 31^st^ 2010, more than 127,000 laboratorial confirmed cases including 800 deaths were reported in the country [4].

Since the H1N1 pandemic in 2009, numerous field investigations and epidemiological studies have investigated the spatial-temporal dynamics, geographic distribution and patient characteristics of the 2009 H1N1 pandemic. Through analyses of a total of 377 H1N1-associated deaths in the United States, Fowlkes et al. demonstrated that the H1N1-associated mortality rate varied substantially in different geographic regions (i.e. highest in Hawaii, New York and Utah) and in different age groups of the infected population (i.e. 76% in patients aged 18–65 years and 9% in patients aged ≥ 65 years) [5]. Similar geographic region- and patient age-dependent incidence of H1N1 infection and subsequent mortality have also been demonstrated in South America [6], and Australia [7]. In addition, the susceptibility to H1N1 infection may vary in different races; Wenger et al. shown that Alaska Native people and Asian/Pacific Islanders (A/PI) were 2–4 times more likely to be infected by H1N1 virus and hospitalized than white Caucasians [8].

To date, little is known regarding the spatio-temporal characteristics of the 2009 pandemic H1N1 as well as factors affecting the recovery of the infected patients in mainland China, especially for the initial cases, who were younger and more mobile than general population. The present study was therefore conducted to investigate the geographic distribution of H1N1 infection in the 2009 pandemic as well as the risk factors for adverse patient outcome after initial H1N1 virus infection in mainland China. Our goal was to generate solid scientific bases for adequate preparedness for potential H1N1 pandemic in the future.

## Results

### Basic characteristics/denigraphics of the patients

Given the unique nature of 2009 pandemic H1N1, both suspected and laboratory confirmed cases had to be reported to the Chinese Centre for Disease Control and Prevention (CCDC). During May 7^th^ and August 12^th^, 2009, the number of reported laboratory-confirmed H1N1 cases was 2668. In this study, we analyzed the data of 885 adult hospitalized patients, accounting for 68.6% of the total confirmed cases. The major demographic and clinical characteristics of these patients are summarized in Table 1.

**Table 1:** Baseline Characteristics of Confirmed Adult Cases.

Characteristic	Count (n = 885)	%
Male sex	486/885	54.9
Age range from 15 to 40	745/885	84.2
Chronic pulmonary disease	25/882	2.8
Delay to hospital admission (≤2 days)	590/769	66.7
General symptoms	852/885	96.3
Respiratory symptoms	833/885	94.1
Pharyngeal abnormality	822/885	92.9
Tonsil abnormality	333/885	37.6
Abnormal white blood cells count	164/849	18.5
Complication	108/839	12.2
Antiviral medication	684/885	77.3
Recovery	815/885	92.1

### Clinical outcomes for the infected patients

Of the 885 hospitalized adult cases, 590 (66.7%) experienced a delay≤2 days in hospital admission, 815 (92.1%) completely recovered and were free of H1N1-associated symptoms on discharge whereas 70 (7.9%) were censored (not recovered when left hospitals). The median time from illness onset to hosptial discharge was 7 days (inter-quartile range 2–22).

The analyses using the proportional hazards model showed that gender, time from illness onset to hospital admission, general symptoms and respiratory symptoms were all related to the outcome of the H1N1 patients (Table 2). Compared with male patients, females patients had a lower porpotion of recovery (HR 0.9, 95%CI: 0.7–1.0), but the differnence was not statistically significant. Delay in hospital admission (longer than 2 days) decreased the probability of recovery (HR 0.6, 95%CI: 0.5–0.7). General symptoms and respiratory symptoms were adversely associated with the rate of recovery in hospital with HR of 0.7 (95%CI: 0.5–1.0) and 0.3 (95%CI: 0.2–0.4), respectively.

**Table 2:** Risk Factors for Recovery in Confirmed Adult Cases.

Variable	*β*	*SE*	Wald χ^2^	*P*-value	*RR* (95% *CI*)
Gender	−0.146	0.078	3.468	0.063	0.864 (0.741–1.008)
time to hospital admission	−0.591	0.094	39.166	<0.001	0.554 (0.460–0.667)
General symptoms	−0.397	0.205	3.740	0.053	0.673 (0.450–1.005)
Respiratory symptoms	−1.263	0.172	53.819	<0.001	0.283 (0.202–0.396)

**Figure 1:**
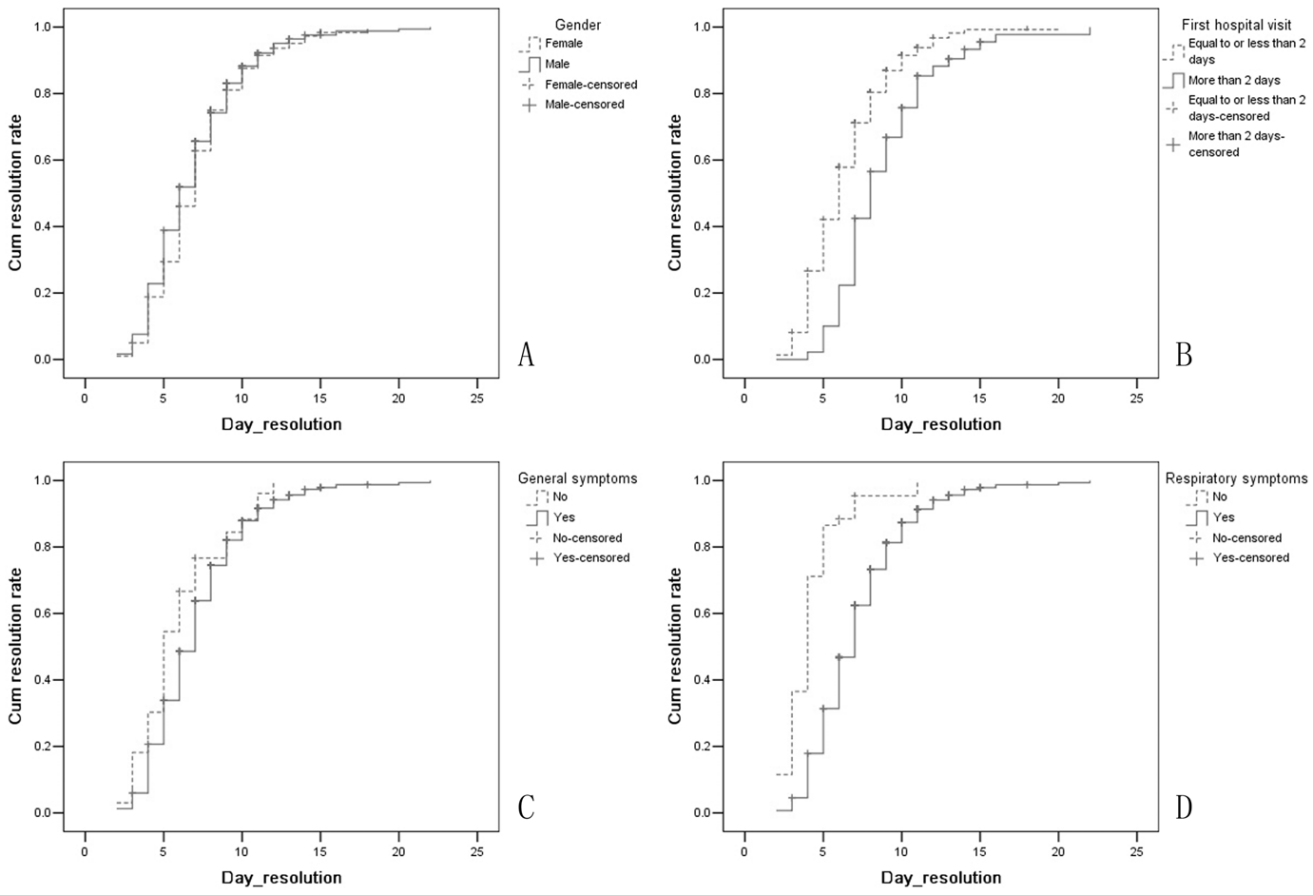
Line graphs, showing results of univariate Cox regression analyses of the cumulative resolution rate curve in different subgroups. A: Male vs Female; B: Delayed hospital admission vs in-time hospital admission; C: With general symptoms vs Without general symptoms; D: With respiratory symptoms vs Without respiratory symptoms.

Showed in Figure 1 are the cumulative resolution rates. It is apparent that the cumulative resolution rate was lower in patients with delayed hospital admission than in those promptly admitted (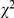  = 60.978, *P*<0.001), and lower in patients with general symptoms (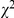  = 3.977, *P* = 0.046) or respiratory symptoms (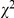  = 100.261, *P*<0.001) than in those without corresponding symptoms. No significant difference was observed between genders (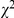  = 1.634, *P* = 0.201).

### Geographic Distribution

**Figure 2:**
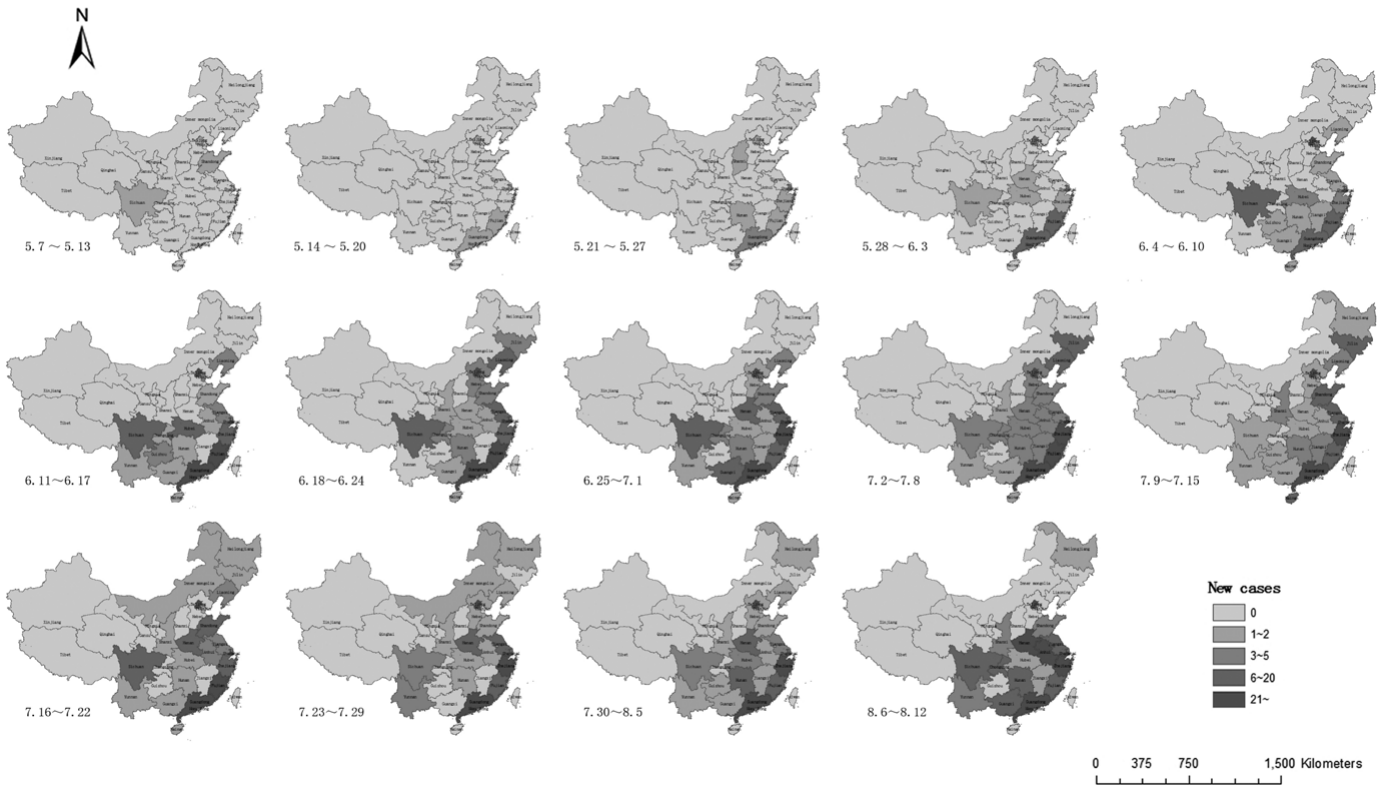
Weekly changes in the geographic distribution of reported H1N1 infection in mainland China between May 7, 2009 and August 12, 2009.

The weekly reported new cases of confirmed H1N1 infection in different geographic regions of mainland China during May 7^th^ to August 12^th^, 2009 are presented in Figure 2. A total of 26 provinces or cities had confirmed cases of infection, among which Beijing city had the highest number of infection (670 cases), followed by Guangdong province(628 cases), Shanghai city(318 cases), Zhejiang province (214 cases), and Fujian province(195 cases).

The number of new cases increased slowly in May 2009, but rapidly between June and August. In May, 87.3% of the cases were imported ones, while the domestic cases (67.3%) became dominant between June and August.

Most of the provinces had reported laboratory-confirmed cases by August. However, the cases were more likely to occur in the southeast coastal areas or areas that serve as major transport stations with relatively prosperous economy, convenient transport system and greater population.

## Discussion

In this study, patients with leukocyte and neutrophils abnormalities as well as patients undergoing antivirus treatment were excluded from the Cox regression model. In seasonal influenza, cough, mild leukopenia, and mild C-reactive protein elevation are relatively common clinical manifestations [9]. Pathologically, the H1N1 virus has features similar to those encountered in the infection with other highly virulent influenza A viruses such as the 1918 H1N1 and H5N1 viruses [10]. However, as the disease was generally a mild procedure, typical inflammation molecules might not be qualified to serve as diagnostic markers. For patients with complications, the secondary bacterial infection which causes elevation in leucopenia and C-reactive protein is most likely to occur. The widely used antivirus treatment like oseltamivir might increase the proportion of oseltamivir-resistant influenza A. Therefore, alternative treatment and vaccination (although, low coverage) are highly recommended.

It has been documented that the adverse outcome, particularly a severe form (i.e. entering ICU or death), after influenza infection is associated with numerous risk factors, including age and underlying medical conditions of the patients. Nolan et al. in a recent study on pandemic or seasonal influenza have observed that infants, the elderly and people suffering from chronic diseases are of high risks for adverse outcome [11]; some other authors have demonstrated significant adverse clinical outcome in patients aged ≥20 years and patients delayed in hospital admission [12], [13]. Nevertheless, only adult hospitalized cases were included in the present study.

With the fast-growing public transportation and increasing socio-economic activities, population mobility has become a concerned problem in the prevention of infectious diseases. Ever since the outbreak of SARS, the central role that Geographic Information System (GIS) plays in the early detection and rapid response to infectious diseases has become evident to researchers as well as policy markers [14], [15]. However, how to integrate GIS into the traditional epidemiological model is a key challenge[16]. Use of GIS in our analyses in this study showed that the incidence of H1N1 infection in the 2009 pandemic in mainland China was higher in the coastal provinces in the southeast as well as in Beijing and Shanghai, two of the most populous cities in the country, where the infection occurred in clusters in the initial phase, followed by spread to adjacent provinces. Moreover, the analyses also demonstrated that within some of the provinces with confirmed cases, the number of H1N1 infection varied widely as well. In most cities and districts, there was still no H1N1 confirmed cases, the overall spread level of H1N1 virus was still regional. Nevertheless, our analyses on the H1N1 spread and the geographic distribution were qualitative only; further quantitative analyses such as correlation or Poisson regression are needed to test the hypothesis that the H1N1 virus spreads more quickly in the crowd area with more convenient travel system.

The limitation of this study is that it only enrolled the adult hospitalized patients. This study included no pediatric cases and therefore was unable to assess the differences in risk factors for adverse patient outcome between children and adults. In the initial phase of H1N1 infection in mainland China, hospital admission was required of every confirmed case. The information on the confirmed cases was from the Chinese Infectious Disease Surveillance System. Sub-clinical infection was common in H1N1 infection. People with mild symptoms might not be included in the Chinese Infectious Disease Surveillance System, possibly causing bias in our analyses. Further studies were needed in the mathematical model and simulation for the transmission characteristics.

Despite the limitations, this study through retrospectively analyses characterized clinical features, disease course, and hospitalization period of the 2009 pandemic H1N1 in mainland China. In adult H1N1 patients, age, chronic pulmonary disease and the appearance of general symptoms, delayed hospital admission and the appearance of respiratory symptoms but not gender were independent risk factors for adverse outcome. Our study also documented the spatial and temporal characteristics of the 2009 pandemic H1N1 at its early stage in mainland China.

## Materials and Methods

### Ethics Statement

The study was approved by ethic committees of both Capital Medical University in Beijing, China and the Chinese Centre for Disease Control and Prevention.

### Data Collection

Data on a total of 885 adult confirmed H1N1 cases from the CCDC. A confirmed case was defined as a person with influenza-like clinical manifestations and positive laboratory test for H1N1 virus, as assessed with the use of a reverse-transcriptase-polymerase-chain-reaction (RT-PCR) assay. Those patients who were still asymptomatic but positive for the RT-PCR assay were quarantined. The cases included in this study were those reported in the early phase of the epidemic between May 7^th^ and August 16^th^, 2009 to the CCDC by sub-CCDC offices after face to face interviews with the individuals of suspected infection.

The case report form was used, which included the following information: gender, age, chronic pulmonary disease history, time from onset to hospital admission, general symptoms (at least one of the following: body temperature≥37.5°C, dizziness, headache, chill, myalgia, arthralgia, weakness, anorexia, and/or conjunctivitis), respiratory symptoms (at least one of the following: runny nose, nasal obstruction, sneezing, dry cough, sputum, sore throat, pharyng itching, shortness of breath, and/or chest pain), pharyngeal abnormality, tonsil abnormality, white blood cells count, complication(s) (at least one of pneumonia, liver function impairment, and/or myocardic injury), antiviral medication and outcome.

### Clinical Outcome

Recovery was defined as resolution of symptoms as well as negative test results for H1N1 virus RNA for two consecutive days in throat swabs.

### Statistical Analysis

Survival analysis was used to evaluate adverse factors for recovery (days from illness onset to recovery) in the early phase of H1N1 epidemic. Potential risk factors assessed included gender, age, chronic pulmonary disease, time from onset to hospital admission, general symptoms, respiratory symptoms, pharyngeal abnormality, tonsil abnormality, white blood cells count, complication and antiviral medication.

The parameters of the recovery model were estimated by backward stepwise likelihood ratio test in proportional hazards model. Censored patient was considered as not recovered. Two-sided *P* values and 95% confidence interval of the parameters were calculated. The cumulative resolution rate was analyzed using Kaplan-Meier analysis and log-rank test and compared between subgroups of patients. The subgroups were based on gender, time from onset to hospital admission, general symptoms or respiratory symptoms. Geo-statistical maps were used to characterized the spatial and temporal distribution of weekly confirmed cases during May 7^th^ and August 12^th^. Longitude and latitude coordinates of each province were identified from a national GIS database. The procedure was fulfilled in a GIS (ArcInfo 9.2, ESRI, Redlands, CA) by overlaying a national vector map.

All statistical analyses were conducted with the SPSS 13.0 software for Windows (SPSS Inc, Chicago, IL, USA).

## References

[1] WHO. Current WHO phase of pandemic alert.. http://www.who.int/csr/disease/avian_influenza/phase/en/index.html.

[2] WHO. Pandemic (H1N1) 2009 - update 96.. http://www.who.int/csr/don/2010_04_16/en/index.html.

[3] Ministry of Health of the People's Republic of China. The third version of the clinical guideline for the pandemic H1N1 influenza.. http://www.moh.gov.cn/publicfiles/business/htmlfiles/mohwsyjbgs/s9990/200905/40478.htm.

[4] Ministry of Health of The People's Republic of China. National influenza H1N1 prevention and control-update March (2010). http://www.moh.gov.cn/publicfiles/business/htmlfiles/mohwsyjbgs/s3578/201004/46480.htm.

[5] Fowlkes AL, Arguin P, Biggerstaff MS, Gindler J, Blau D (2011). Epidemiology of 2009 pandemic influenza A (H1N1) deaths in the United States, April-July 2009.. Clin Infect Dis.

[6] Chowell G, Viboud C, Munayco CV, Gomez J, Simonsen L (2011). Spatial and temporal characteristics of the 2009 A/H1N1 influenza pandemic in Peru.. PLoS One.

[7] Khandaker G, Dierig A, Rashid H, King C, Heron L (2011). Systematic review of clinical and epidemiological features of the pandemic influenza A (H1N1) 2009.. Influenza Other Respi Viruses.

[8] Wenger JD, Castrodale LJ, Bruden DL, Keck JW, Zulz T (2011). 2009 Pandemic influenza A H1N1 in Alaska: temporal and geographic characteristics of spread and increased risk of hospitalization among Alaska Native and Asian/Pacific Islander people.. J Infect Dis.

[9] Jeong I, Lee CH, Kim DK, Chung HS, Park SW (2010). Mild form of 2009 H1N1 influenza infection detected by active surveillance: Implications for infection control.. Am J Infect Control.

[10] Guarner J, Falcón-Escobedo R (2009). Comparison of the pathology caused by H1N1, H5N1, and H3N2 influenza viruses.. Arch Med Res.

[11] Nolan T, McVernon J, Skeljo M, Richmond P, Wadia U (2010). Immunogenicity of a Monovalent 2009 influenza A (H1N1) vaccine in infants and children.. JAMA.

[12] Campbell A, Rodin R, Kropp R, Mao Y, Hong Z (2010). Risk of severe outcomes among patients admitted to hospital with pandemic (H1N1) influenza.. CMAJ.

[13] Seema J, Laurie K, Anna MB, Ann MS, Stephen RB (2009). Hospitalized patients with 2009 H1N1 influenza in the United Stats, April-June 2009.. N Engl J Med.

[14] Rogers DJ, Randolph SE (2003). Studying the global distribution of infectious diseases using GIS and RS.. Nat Rev Microbiol.

[15] Boulos MN (2004). Descriptive review of geographic mapping of severe acute respiratory syndrome (SARS) on the Internet.. Int J Health Geogr.

[16] Graham AJ, Atkinson PM, Danson FM (2004). Spatial analysis for epidemiology.. Acta Trop.

